# Diesterified Nitrone Rescues Nitroso-Redox Levels and Increases Myocyte Contraction Via Increased SR Ca^2+^ Handling

**DOI:** 10.1371/journal.pone.0052005

**Published:** 2012-12-27

**Authors:** Christopher J. Traynham, Steve R. Roof, Honglan Wang, Robert A. Prosak, Lifei Tang, Serge Viatchenko-Karpinski, Hsiang-Ting Ho, Ira O. Racoma, Dominic J. Catalano, Xin Huang, Yongbin Han, Shang-U Kim, Sandor Gyorke, George E. Billman, Frederick A. Villamena, Mark T. Ziolo

**Affiliations:** 1 Department of Physiology and Cell Biology, The Ohio State University, Columbus, Ohio, United States of America; 2 Department of Pharmacology, Davis Heart and Lung Research Institute, The Ohio State University, Columbus, Ohio, United States of America; Brigham & Women’s Hospital - Harvard Medical School, United States of America

## Abstract

Nitric oxide (NO) and superoxide (O_2_^−^) are important cardiac signaling molecules that regulate myocyte contraction. For appropriate regulation, NO and O_2_^.−^ must exist at defined levels. Unfortunately, the NO and O_2_^.−^ levels are altered in many cardiomyopathies (heart failure, ischemia, hypertrophy, etc.) leading to contractile dysfunction and adverse remodeling. Hence, rescuing the nitroso-redox levels is a potential therapeutic strategy. Nitrone spin traps have been shown to scavenge O_2_^.−^ while releasing NO as a reaction byproduct; and we synthesized a novel, cell permeable nitrone, 2–2–3,4-dihydro-2H-pyrrole 1-oxide (EMEPO). We hypothesized that EMEPO would improve contractile function in myocytes with altered nitroso-redox levels. Ventricular myocytes were isolated from wildtype (C57Bl/6) and NOS1 knockout (NOS1^−/−^) mice, a known model of NO/O_2_^.−^ imbalance, and incubated with EMEPO. EMEPO significantly reduced O_2_^.−^ (lucigenin-enhanced chemiluminescence) and elevated NO (DAF-FM diacetate) levels in NOS1^−/−^ myocytes. Furthermore, EMEPO increased NOS1^−/−^ myocyte basal contraction (Ca^2+^ transients, Fluo-4AM; shortening, video-edge detection), the force-frequency response and the contractile response to β-adrenergic stimulation. EMEPO had no effect in wildtype myocytes. EMEPO also increased ryanodine receptor activity (sarcoplasmic reticulum Ca^2+^ leak/load relationship) and phospholamban Serine16 phosphorylation (Western blot). We also repeated our functional experiments in a canine post-myocardial infarction model and observed similar results to those seen in NOS1^−/−^ myocytes. In conclusion, EMEPO improved contractile function in myocytes experiencing an imbalance of their nitroso-redox levels. The concurrent restoration of NO and O_2_^.−^ levels may have therapeutic potential in the treatment of various cardiomyopathies.

## Introduction

Despite recent advances in treatment strategies, heart failure (HF) is a growing epidemic that still presents with poor clinical prognosis. Thus, the development of new therapeutic agents is of vital importance. Recently, therapies have been developed to target superoxide (O_2_^.−^) or nitric oxide (NO) [1]. For both of these signaling molecules to appropriately regulate myocyte contraction, they must exist at defined levels [2]. The levels of these reactive nitrogen and oxygen species (RNS, ROS) depend upon their production and scavenging. In disease, the O_2_^.−^ and NO levels are altered and these imbalances contribute to both the contractile dysfunction and adverse remodeling observed in various cardiomyopathies. Specifically, O_2_^.−^ production is increased in heart failure (HF) via NADPH oxidase, xanthine oxidase, and/or mitochondria; while O_2_^.−^ degradation is decreased via a reduction in superoxide dismutase activity [3]–[6]. In hypertrophy, there is an increased production of O_2_^.−^ due to uncoupling of NOS3 [7] and during ischemia/reperfusion (I/R) injury there is a burst in O_2_^.−^ production from mitochondria [8]. As a result, antioxidants have been developed and used as potential therapeutics. Unfortunately, in a clinical trial, the XO inhibitor oxypurinol did not lead to clinical benefits in HF patients [9]. This type of therapy may not have been beneficial since reducing O_2_^.−^ levels by itself will not restore the altered nitroso levels because there are also changes in NO bioavailability [10]. For example, NOS1 is translocated and NOS2 expression is increased in HF, NOS3 becomes uncoupled during hypertrophy, and NOS2 expression also occurs with I/R injury [7], [11]–[13]. Thus, a therapy is needed that will restore both O_2_^.−^ and NO levels.

**Figure 1:**
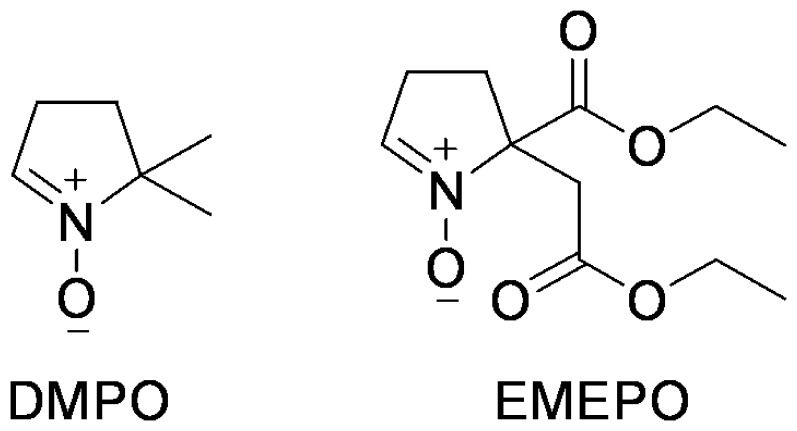
Structure of DMPO and EMEPO.

Spin traps have been used as reagents to detect and to identify transient radicals including O_2_^.−^ using electron paramagnetic resonance spectroscopy in chemical and biological systems. Nitrone spin traps, 5,5-dimethylpyrroline N-oxide (DMPO), α-phenyl-tert-butyl-nitrone (PBN) and its sulfonyl derivative, NXY-059, have shown pharmacological activity against I/R injury in the heart and brain [14]. With their NO-releasing capabilities [15], nitrones have also been shown to protect against stroke [16] and improve cerebral blood flow [17] in animal models. Our recent work has demonstrated that DMPO is cardioprotective in hearts undergoing I/R injury [14]. Although nitrones have shown cardioprotective effects, the molecular mechanism of their action is not fully understood. Specifically, their role in rescuing O_2_^.−^ and NO levels, myocyte contraction, and particularly Ca^2+^ handling, are not known. A novel ester derivative of DMPO, 2-(2-ethoxy-2-oxoethyl)-2-(ethoxycarbonyl)-3,4-dihydro-2H-pyrrole 1-oxide (EMEPO) (Figure 1), was therefore synthesized allowing for permeation of the cell membrane. Thus, EMEPO is expected to impart enhanced cellular pharmacological activity compared to other treatments.

Previously, our laboratory and others have studied the effects of neuronal nitric oxide synthase knockout (NOS1^−/−^) on the heart’s contractile function. Ventricular myocytes from NOS1^−/−^ mice exhibit decreased basal contraction (although increased basal contraction has also been reported), slowed relaxation, a blunted force-frequency response, and a decreased functional response to β-adrenergic (β-AR) stimulation compared to wild-type (WT) myocytes [18]–[24]. NOS1^−/−^ myocytes also have increased O_2_^.−^ levels and decreased NO bioavailability, thus mimicking the altered nitroso-redox levels often observed in disease states [25]–[28]. Due to these characteristics, we hypothesize that EMEPO will improve contractile function in NOS1^−/−^ myocytes via the rescuing of both O_2_^.−^ and NO levels.

## Methods

An expanded Methods section is available in the Supplementary Text S1. In brief, adult ventricular myocytes were isolated from mice (NOS1^−/−^, C57Bl/6- WT) and canines (control, post-myocardial infarction). O_2_^.−^ levels were measured in myocyte homogenates via lucigenin-enhanced chemiluminescence. NO levels were measured in myocytes via DAF-FM diacetate. Myocyte contraction was evaluated by simultaneous measurement of cell shortening, via edge detection, and calcium transients, via epifluorescence (Fluo4-AM). Sarcoplasmic reticulum (SR) Ca^2+^ leak was measured as the tetracaine (ryanodine receptor (RyR) inhibitor)-induced shift in diastolic [Ca^2+^]_i_ normalized to its corresponding SR Ca^2+^ load. Experiments were performed at room temperature. Western blots were utilized to measure phospholamban (PLB) Serine16 phosphorylation in myocyte homogenates.

## Results

### EMEPO Rescues O_2_^.−^ and NO Levels in NOS1^−/−^ Myocytes

**Figure 2:**
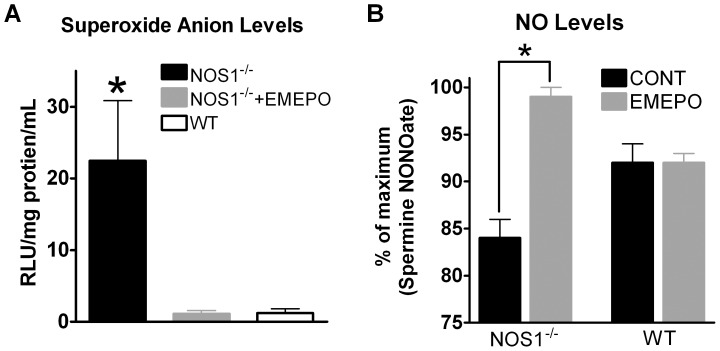
EMEPO decreases O_2_^.−^ levels and increases NO levels in NOS1^−/−^ myocytes. A: Summary data (mean±s.e.m.) of O_2_^.−^ levels in WT and NOS1^−/−^ myocytes. * P<0.05 NOS1^−/−^ vs. WT and NOS1^−/−^+EMEPO. n = 3–4 hearts/group. B: Summary data (mean±s.e.m.) of NO levels in NOS1^−/−^ and WT myocytes (±EMEPO). * P<0.05 vs. -EMEPO. n = 13–20 myocytes per group.

Previous studies have shown that O_2_^.−^ levels are increased and NOS1 activity is decreased (and thus NO bioavailability) in NOS1^−/−^ myocytes [25]–[28]. Thus, we examined if 1 mM EMEPO was able to correct the aberrant O_2_^.−^ and NO levels. NOS1^−/−^ myocytes had significantly higher O_2_^.−^ levels compared to WT myocytes (22.4±8.4 vs.1.2±0.6 RLU, P<0.05, Figure 2A), which was decreased with EMEPO (1.1±0.4 RLU, P<0.05 vs. +EMEPO, Figure 2A). There was no difference in O_2_^.−^ levels between NOS1^−/−^+EMEPO vs. WT myocytes, suggesting near complete O_2_^.−^ scavenging. We also measured NO levels in NOS1^−/−^ myocytes. As shown in Figure 2B, NOS1^−/−^ myocytes incubated with 1 mM EMEPO had increased NO bioavailability vs. control NOS1^−/−^ myocytes (i.e. no EMEPO incubation) (84±2 vs. 99±1% of maximum DAF fluorescence; P<0.05). Furthermore, we also observed that EMEPO incubation had no effect on NO levels in WT myocytes (92±2 vs. 92±1% of maximum DAF fluorescence; P = NS). Thus, EMEPO increased NO bioavailability. These data suggest that EMEPO is able to rescue nitroso-redox levels in NOS1^−/−^ myocytes by decreasing O_2_^.−^ and increasing NO levels.

### EMEPO Increases Contractile Function in NOS1^−/−^ Myocytes

**Figure 3:**
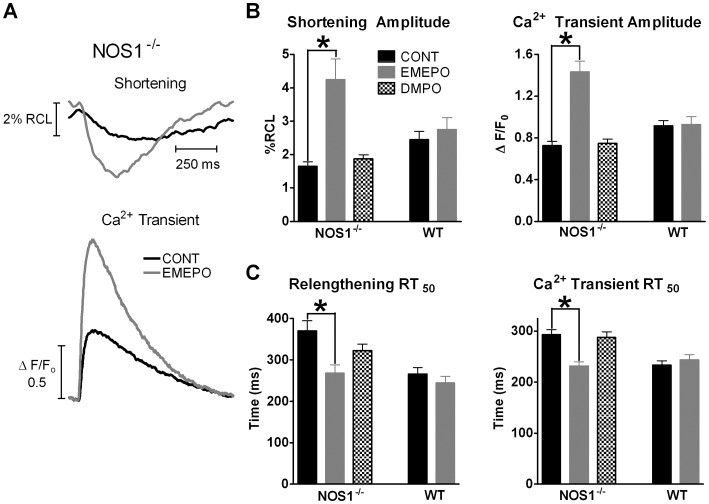
EMEPO increases contraction in NOS1^−/−^ myocytes with no effect in WT myocytes. A: Individual, steady-state cell shortening (top) and Ca^2+^ transient (bottom) traces measured in NOS1^−/−^ (CONT, black) and EMEPO incubated NOS1^−/−^ (EMEPO, gray) myocytes. B: Summary data (mean±s.e.m.) of the effects of EMEPO and DMPO on shortening (left) and Ca^2+^ transient (right) amplitudes. C: Summary data (mean±s.e.m.) of the effects of EMEPO and DMPO on rate of relaxation (left) and [Ca^2+^]_i_ decline (right) measured as time to 50% relaxation (RT_50_). ^*^ P<0.05 vs. control. n = 6 cells/3 hearts for NOS1^−/−^, n = 18 cells/3 hearts for NOS1^−/−^+EMEPO, and n = 21 cells/3 hearts for NOS1^−/−^+DMPO, n = 25 cells/5 hearts for WT, n = 22 cells/3 hearts for WT+EMEPO.

Since 1 mM EMEPO rescued O_2_^.−^ and NO levels, we then determined the effects of 1 mM EMEPO on murine ventricular myocyte basal contraction (stimulation frequency of 1.0 Hz). Shown in Figure 3A are representative shortening and Ca^2+^ transient traces in the presence or absence of EMEPO. As shown in Figure 3B, NOS1^−/−^ myocytes that were incubated with EMEPO had significantly increased shortening (1.7±0.1 vs. 4.3±0.6%RCL; P<0.05) and Ca^2+^ transient (0.7±0.1 vs. 1.4±0.1 ΔF/F_0_; P<0.05) amplitudes compared to control NOS1^−/−^ myocytes (i.e., not incubated with EMEPO). Furthermore, EMEPO was able to enhance the rate of relaxation measured as the time to 50% relaxation (RT_50_) (relengthening RT_50_∶370±25 vs. 268±20 ms; P<0.05, Figure 3C) and the Ca^2+^ transient decline (RT_50_∶294±10 vs. 232±8 ms; P<0.05, Figure 3C). Our observed effects of EMEPO on contraction were similar in experiments performed early or late after incubation, suggesting the effects of EMEPO are not reversible at these time points. We also compared the effects of incubating NOS1^−/−^ myocytes with 0.25 mM and 0.5 mM EMEPO. While we did observe positive inotropic and lusitropic effects with 0.25 mM and 0.5 mM EMEPO (data not shown), our greatest effect was with 1 mM EMEPO. Thus, we used 1 mM EMEPO for this study. These data suggest that EMEPO can improve inotropy and lusitropy in NOS1^−/−^ myocytes.

We also determined the effect of EMEPO on WT myocyte contractile function, which possess normal O_2_^.−^ and NO levels. Shown in Figure 3B, EMEPO had no effect on WT myocyte shortening (2.4±0.3 vs. 2.8±0.4%RCL, P = NS) and Ca^2+^ transient (0.9±0.1 vs. 0.9±0.1 ΔF/F_0_, P = NS) amplitudes. There was also no effect of EMEPO on relengthening (RT_50_∶266±15 vs. 244±16 ms, P = NS; Figure 3C) or the Ca^2+^ transient decline (RT_50_∶234±9 vs. 244±10 ms, P = NS; Figure 3C). These data suggest EMEPO does not affect contractile function in myocytes which have normal O_2_^.−^ and NO levels.

We also determined the effects of EMEPO on diastolic Ca^2+^ levels (measured as the Fluo-4 F_0_ value). Consistent with previous results [24], we did not observe a difference in diastolic Ca^2+^ levels between WT and NOS1^−/−^ myocytes (0.35±0.04 vs 0.36±0.04). EMEPO did not affect diastolic Ca^2+^ levels in WT or NOS1^−/−^ myocytes (0.36±0.03 vs 0.33±0.04).

The effect of DMPO, the parent molecule of EMEPO, was also evaluated on NOS1^−/−^ myocyte contraction. DMPO lacks ester groups and should poorly permeate the cell membrane Shown in Figure 3B–C, DMPO had no effect on NOS1^−/−^ myocyte shortening amplitude (1.8±0.1%RCL), Ca^2+^ transient amplitude (0.8±0.1 ΔF/F_0_), relengthening (RT_50_∶323±15 ms), or Ca^2+^ transient decline (RT_50_∶286±10 ms). These data provide evidence that EMEPO, an intracellularly targeted nitrone, exerted unique effects on NOS1^−/−^ contractile function.

**Figure 4:**
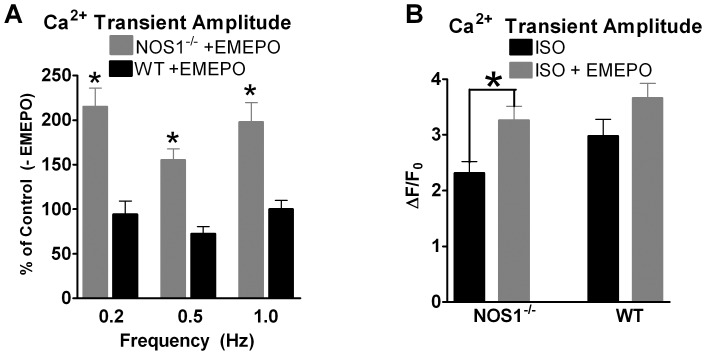
EMEPO increases FFR and potentiates the β-AR response in NOS1^−/−^ myocytes. A: Summary data (mean±s.e.m.) of the effect of EMEPO in NOS1^−/−^ and WT myocytes at various stimulation frequencies. ^*^ P<0.05 vs. NOS1^−/−^ -EMEPO. n = 37 cells/5 hearts for WT+EMEPO and 37cells/6 hearts for NOS1^−/−^+EMEPO (gray). n = 5–8 myocytes/3 hearts for WT and NOS1^−/−^ - EMEPO. B: Summary data (mean±s.e.m.) of the effect of EMEPO on β-AR stimulated Ca^2+^ transient amplitudes in NOS1^­/−^ and WT myocytes. ^*^ P<0.05 NOS1^−/−^ +ISO vs NOS1^−/−^ +ISO/EMEPO. n = 13 cells/5 hearts for NOS1^−/−^ +ISO, n = 10 cells/4 heart for NOS1^−/−^ +ISO/EMEPO, n = 16 cells/6 hearts for WT +ISO, n = 22 cells/6 hearts for WT +ISO/EMEPO.

Since NOS1^−/−^ myocytes have a blunted FFR [19], [21], we extended our contractile studies to examine if EMEPO could enhance FFR. Under our experimental conditions (isolated myocyte, room temperature, stimulation frequencies below 1 Hz), we observed a flat or slightly negative FFR in WT myocytes (data not shown). In NOS1^−/−^ myocytes, contraction (shortening and Ca^2+^ transient amplitudes) is reduced at all frequencies compared to WT (data not shown). Shown in Figure 4A, EMEPO increased Ca^2+^ transient amplitudes in NOS1^−/−^ myocytes at all frequencies tested (0.2 Hz: 215±21% of 0.2 Hz –EMEPO; 0.5 Hz: 155±12% of 0.5 Hz –EMEPO, and 1.0 Hz: 198±21% of 1.0 Hz –EMEPO; all P<0.05 vs NOS1^−/−^ –EMEPO). EMEPO had no effect at any frequency tested in WT myocytes (0.2 Hz: 94±15% of 0.2 Hz –EMEPO; 0.5 Hz: 72±15% of 0.5 Hz –EMEPO, and 1.0 Hz: 100±9% of 1.0 Hz –EMEPO). Thus, EMEPO increased NOS1^−/−^ contraction at all the frequencies tested to or above WT levels. However, NOS1^−/−^ myocytes treated with EMEPO still exhibited the flat/slightly negative FFR. These data suggest that the positive inotropic effects of EMEPO occur across various stimulation frequencies to increase contraction.

NOS1^−/−^ myocytes additionally exhibit a decreased functional response to β-AR stimulation [18], [21]. Therefore, we also determined the effect of EMEPO on β-AR stimulated contraction in NOS1^−/−^ and WT myocytes. Shown in Figure 4B, EMEPO incubation significantly increased β-AR stimulated Ca^2+^ transient amplitude in NOS1^−/−^ myocytes compared to non-incubated NOS1^−/−^ myocytes (2.3±0.2 vs. 3.3±0.2 ΔF/F_0_; P<0.05). There was no difference in β-AR stimulated Ca^2+^ transient amplitude in EMEPO incubated WT myocytes compared to control WT myocytes (3.0±0.3 vs. 3.7±0.3, ΔF/F_0_; P = 0.08). These data suggest EMEPO can potentiate β-AR stimulated contractile function in NOS1^−/−^ ventricular myocytes.

### EMEPO Produces Greater Contractile Effects than a O_2_^.−^ Scavenger or a NO Donor

**Figure 5:**
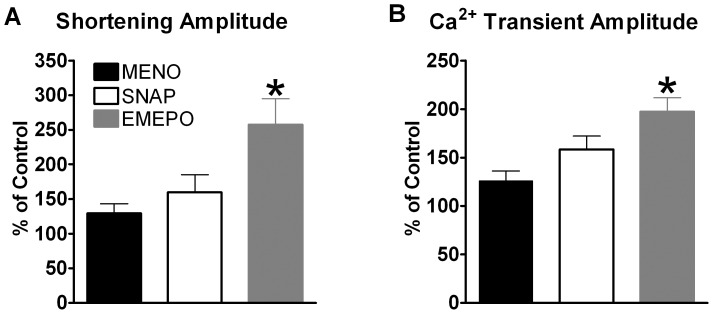
Larger increase in contraction with EMEPO compared to a superoxide scavenger or NO donor (SNAP) in NOS1^−/−^ myocytes. Summary data (mean±s.e.m.) of the effects of MENO, SNAP, and EMEPO on shortening (A) and Ca^2+^ transient (B) amplitudes. ^*^ P<0.05 EMEPO vs. MENO and SNAP. n = 17 cells/5 hearts for NOS1^−/−^ +MENO, n = 25 cells/5 hearts NOS1^−/−^ +SNAP, and n = 18 cells/3 hearts for NOS1^−/−^ +EMEPO.

EMEPO was synthesized to be both a O_2_^.−^ scavenger and a NO donor. Therefore, we next determined how EMEPO’s functional effects compared to those of a cell-permeable O_2_^.−^ scavenger (Methyl-ester Nitroxide, MENO) and a NO donor (SNAP). MENO, SNAP, and EMEPO all significantly increased NOS1^−/−^ myocyte contraction (data not shown). However, as shown in Figure 5, EMEPO incubated myocytes exhibited a larger percent increase in shortening (MENO: 129±14; SNAP: 159±26 vs. EMEPO: 258±37% of control, P<0.05 vs MENO and SNAP) and Ca^2+^ transient (MENO: 126±10; SNAP: 158±14 vs. EMEPO: 197±14% of control, P<0.05 vs MENO and SNAP) amplitudes. These data suggest that EMEPO produces functional effects unique from other redox treatments.

### Mechanisms Responsible for the EMEPO-dependent Increase in Contraction

**Figure 6:**
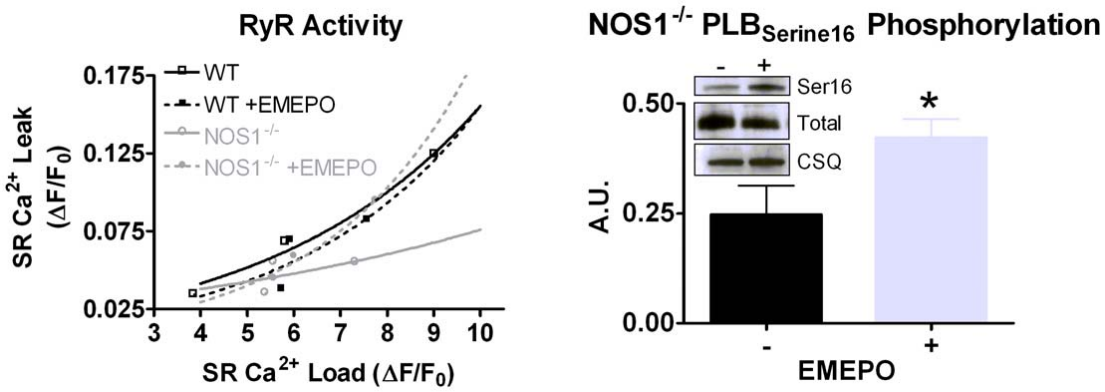
EMEPO increases RyR activity and PLB Serine_16_ phosphorylation in NOS1^−/−^ myocytes. A: Plot of the SR Ca^2+^ leak/load relationship in NOS1^−/−^ and WT myocytes. n = 10 cells/6 hearts for NOS1^−/−^, and n = 19 cells/6 hearts NOS1^−/−^ +EMEPO, n = 9 cells/4 hearts for WT, n = 16 cells/5 hearts for WT +EMEPO. B: Summary data (mean±s.e.m.) of EMEPO’s effect on Serine_16_ phosphorylation (normalized to total PLB) in NOS1^−/−^ hearts. n = 4 hearts for NOS1^−/−^ and n = 5 hearts for NOS1^−/−^ +EMEPO. ^*^ P<0.05 vs. control.

Our previous data indicate that the reduced contraction in NOS1^−/−^ myocytes is due to decreased RyR activity and PLB phosphorylation [21], [22]. Therefore, we determined if EMEPO increased contraction via regulation of these protein targets. As seen in Figure 6A, EMEPO was able to restore RyR activity in NOS1^−/−^ myocytes to WT levels (i.e., leftward shift in the SR Ca^2+^ leak/load relationship), and was without effect in WT myocytes. In addition, EMEPO increased PLB Serine16 phosphorylation in NOS1^−/−^ myocytes (0.20±0.07 vs. 0.40±0.04 A.U., P<0.05; Figure 6B). NOS1^−/−^ myocytes had a decreased SR Ca^2+^ load compared to WT myocytes (data not shown), consistent with our and others previous data [19], [21], [22], As expected with our PLB Serine16 phosphorylation data, EMEPO increased SR Ca^2+^ load in NOS1^−/−^ myocytes with no change observed in WT myocytes (132±9% vs 106±6%). Taken together, these data suggest EMEPO increases contraction via regulation of SR Ca^2+^ handling.

### EMEPO Increases Contraction in a Canine Post-myocardial Infarction (MI) Model

We also determined if EMEPO was able to increase myocyte contraction in a post-MI canine model, which exhibits increased ROS levels resulting in altered Ca^2+^ handling [29], [30]. EMEPO significantly increased shortening (5.8±1.4 vs. 15.5±1.7%RCL, P<0.05) and Ca^2+^ transient (0.7±0.1 vs. 1.3±0.1 ΔF/F_0_, P<0.05) amplitudes in post-MI myocytes but had no effect in control canine myocytes (shortening amplitude: 12.4±1.1 vs. 10.9±1.5%RCL; Ca^2+^ transient amplitude: 0.9±0.1 vs. 1.0±0.1 ΔF/F_0_). In addition, EMEPO accelerated relengthening (RT_50_∶584±22 vs. 459±21 ms, P<0.05) and Ca^2+^ transient decline (RT_50_∶589±23 vs. 504±26 ms, P<0.05) in post-MI myocytes but had no effect in control canine myocytes (relengthening RT_50_∶495±14 vs. 536±18 ms; Ca^2+^ transient decline RT_50_∶514±21 vs. 476±24 ms). We did not observe any difference in diastolic Ca^2+^ levels in control and MI myocytes (±EMEPO) (control myocytes- cont: 0.55±0.07, EMEPO: 0.49±0.07; MI myocytes- cont: 0.57±0.09, EMEPO: 0.54±0.11). Thus, EMEPO is able to increase contraction in a disease model with an imbalance of O_2_^.−^ levels.

## Discussion

Our current study demonstrates that a novel O_2_^.−^ scavenger, EMEPO, rescues both O_2_^.−^ and NO levels and improves contractile function in isolated myocytes under conditions of nitroso-redox disequilibrium (i.e., NOS1^−/−^ and post-MI myocytes). Specifically, EMEPO increased basal contraction, FFR, and β-AR stimulated contractile function. EMEPO’s contractile effects were via increased RyR activity and PLB Serine16 phosphorylation. Interestingly, EMEPO also exhibited greater contractile effects compared to other redox treatments (O_2_^.−^ scavenger or NO donor).

### Nitroso-redox Levels in Disease

In healthy myocardium, O_2_^.−^ is produced via XO, mitochondria, and NADPH oxidase, and is rapidly buffered by glutathione and broken down by superoxide dismutase (SOD). However, in diseased myocardium, O_2_^.−^ levels are elevated due to increased production and decreased degradation [3]–[8], [31]. High levels of O_2_^.−^ alter the function of a variety excitation-contraction coupling proteins leading to contractile dysfunction [32], [33]. As a result, antioxidant treatments such as XO and NADPH inhibitors and SOD mimetics have been developed to combat this oxidative damage. These treatments increase contractile function and promote cardioprotection in failing hearts [34], [35]. Interestingly, the success of antioxidants is dependent upon NO bioavailability [36]. That is, the XO inhibitor allopurinol was shown to be ineffective with low levels of NO. This observation becomes important in diseased myocardium since there is also altered NO production. This occurs via the translocation of NOS1 from the SR to the caveolae, uncoupling of NOS3, and/or expression of NOS2 [7], [11], [12]. In fact, it has recently been shown that altered NO bioavailability and higher O_2_^.−^ levels contribute to the cardiac dysfunction present in HF [37]. Thus, altered nitroso-redox levels are a major contributor to the contractile dysfunction and altered remodeling present in many cardiomyopathies [38], making the concurrent rescue of both O_2_^.−^ and NO levels an attractive therapeutic strategy.

### EMEPO Structure and Function

Although cyclic nitrones are structurally simple molecules, they possess rich chemistries and biological properties that make them relevant pharmacological agents. For example, nitrones 1) can act as oxidizing and reducing agents by virtue of their oxidation state [39]; 2) react and scavenge a variety of free radicals [40]; and 3) decompose to NO after addition of O_2_^.−^ [15], [41]. While the non-cell membrane permeable nitrone DMPO showed cardioprotective properties [14], we anticipated that the intracellularly targeted nitrone EMEPO would be more effective in improving myocyte contraction. Thus, EMEPO being both a O_2_^.−^ scavenger and a NO donor [15] may exhibit pharmacological activity against the cardiac mechanical dysfunction caused by disorder of nitroso-redox levels.

### EMEPO Rescues the O_2_^.−^ and NO Levels and Increases Contraction in NOS1^−/−^ Myocytes

Genetic deletion of NOS1 leads to decreases in both NO production and bioavailability ( [28] and Figure 2). Previous studies have also shown that when NOS1 signaling is lost, O_2_^.−^ levels increase [25]–[27]. Increased O_2_^.−^ levels and decreased NO bioavailability (i.e., nitroso-redox disequilibrium) contribute to the decreased basal contractile function, blunted FFR and reduced contractile response to β-AR stimulation in NOS1^−/−^ myocytes [18]–[22]. Additionally, after myocardial infarction (MI), NOS1^−/−^ mice display increased mortality and adverse remodeling due to the imbalanced O_2_^.−^ and NO levels [42], [43]. Thus, NOS1^−/−^ myocytes present an ideal model for a proof of principle study. The intent of our study was to determine if EMEPO could restore O_2_^.−^ and NO levels and improve the contractile dysfunction observed in NOS1^−/−^ myocytes.

As expected, EMEPO, with its unique chemistry, normalized O_2_^.−^ in NOS1^−/−^ myocytes to WT levels as well as increased NO bioavailability (Figure 2). These data reaffirm that EMEPO is novel because it can not only decrease O_2_^.−^ levels but also increase NO levels.

With the rescue of O_2_^.−^ and NO levels, we next determined the effects of EMEPO on myocyte contraction. As hypothesized, EMEPO increased basal Ca^2+^ transient and shortening amplitudes and enhanced the rate of relaxation (Figure 3), increased the FFR (Figure 4A), and the functional response to β-AR stimulation (Figure 4B) in NOS1^−/−^ myocytes. Although EMEPO had no effect in WT myocytes, our data surprisingly suggest a trend toward a potentiated β-AR response. Prior studies have provided evidence that both acute and chronic administration of β-AR agonists can lead to increased O_2_^.−^ production [44]. Thus, we believe that EMEPO’s effect in WT is due to scavenging O_2_^.−^. However, further study is needed to confirm this hypothesis. These contractile effects are unique to the intracellular targeting of EMEPO as DMPO, an extracellular nitrone, was without effect on NOS1^−/−^ myocyte contraction (Figure 3).

We believe that the dramatic improvement of NOS1^−/−^ myocyte contractile function is due to the distinct characteristics of nitrone spin traps (i.e. decreasing O_2_^.−^ and increasing NO levels). That is, rescuing both the O_2_^.−^ and NO levels with EMEPO resulted in significantly greater contraction than those of either the superoxide scavenger MENO or the NO donor SNAP (Figure 5). In fact, MENO only restored NOS1^−/−^ myocyte contraction to WT levels (data not shown) and our previous results showed that SNAP also restored NOS1^−/−^ myocyte contraction to WT levels [22]. However, EMEPO resulted in significantly greater contraction in NOS1^−/−^ compared to WT myocytes (Figure 3). Interestingly, a previous study found that the XO inhibitor allopurinol was able to increase NOS1^−/−^ myocyte shortening but did not affect Ca^2+^ handling [27]. Although, this treatment decreased O_2_^.−^ levels, we believe it was only partially effective in NOS1^−/−^ myocytes because NO signaling was not rescued. However, EMEPO resulted in significantly higher NO levels in NOS1^−/−^ myocytes compared to WT (Figure 2), and increased both [Ca^2+^]_i_ and shortening. Hence, our data suggest that the greater effect of EMEPO can be attributed to an additive effect of enhanced NO signaling and dampened O_2_^.−^ levels.

### EMEPO Increases SR Ca^2+^ Cycling

RyR is an important protein in the heart responsible for the release of Ca^2+^ from the SR and is regulated by a multitude of factors including NO and O_2_^.−^ [32]. That is, S-nitrosylation of RyR results in increased activity [22], while O_2_^.−^ results in decreased or increased activity depending on O_2_^.−^ concentration and duration of exposure [45]. RyR from NOS1^−/−^ hearts have reduced S-nitrosylation levels and increased oxidation [20], [22]. Our data has shown that these effects result in decreased RyR activity, which contributes to the contractile dysfunction [22]. Thus, we investigated if the improved contraction with EMEPO was via increased RyR activity in NOS1^−/−^ myocytes. In a physiologically relevant method, we measured RyR activity using the SR Ca^2+^ leak/load relationship. Consistent with our previous results [22], RyR activity was decreased in NOS1^−/−^ myocytes. Furthermore, incubating NOS1^−/−^ myocytes with EMEPO increased RyR activity to WT levels (Figure 6). Thus, the improvement in contraction in NOS1^−/−^ myocytes is, in part, due to increased Ca^2+^ release from the SR via enhanced RyR activity.

SR Ca^2+^ uptake is also a redox regulated process. For example, reactive nitrogen species (e.g., nitroxyl) can increase SERCA activity by modulating PLB [46]. Furthermore, our and others work has shown that NOS1^−/−^ myocytes have reduced PLB Serine16 phosphorylation resulting in depressed SR Ca^2+^ uptake [21], [24]. This effect has been attributed to a shift in the phosphatase/kinase balance [24], [47]. Thus, we also investigated if EMEPO can increase PLB Serine16 phosphorylation in NOS1^−/−^ myocytes. Our data show that NOS1^−/−^ myocytes incubated with EMEPO had higher PLB phosphorylation levels (Figure 6). We speculate that rescuing O_2_^.−^ and NO levels re-establishes the phosphatase/kinase balance resulting in increased PLB Serine16 phosphorylation. Hence, the improvement in contraction in NOS1^−/−^ myocytes is, in part, due to increased SR Ca^2+^ uptake via increased PLB phosphorylation. Furthermore, we believe that the increased PLB phosphorylation results in the accelerated [Ca^2+^]_i_ kinetics [48], [49], which will ultimately increase SR Ca^2+^ load and, thus, myocyte contraction. Collectively, our RyR activity and PLB phosphorylation data suggest that EMEPO improves contraction via enhanced SR Ca^2+^ cycling.

In addition to RyR and PLB, NO is able to modulate the function of other protein targets, such as Troponin I (TnI) and the L-type Ca^2+^ channel. TnI can be phosphorylated by NO-activated cGMP-dependent protein kinase (PKG) to decrease myofilament Ca^2+^ sensitivity and enhance the rate of relaxation [50].S-nitrosylation of the L-type Ca^2+^ channel via NO will increase Ca^2+^ influx [51] and myocyte contraction. Since NOS1 signaling modulates the L-type Ca^2+^ channel [23], determining if EMEPO modifies other protein end targets warrants further studies.

### EMEPO Increases Contraction in a Post-MI Canine Model

**Figure 7:**
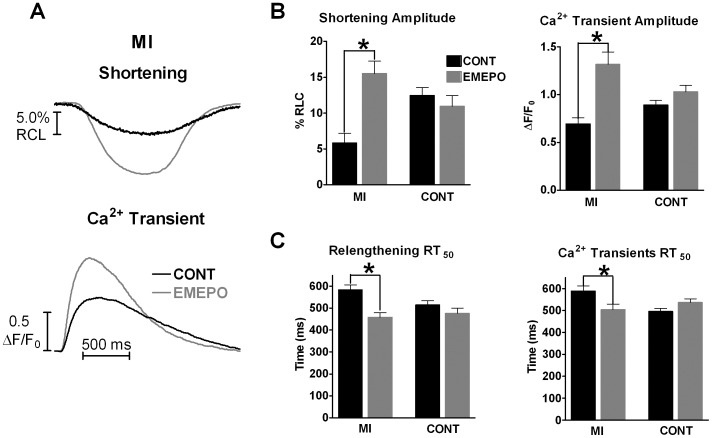
EMEPO increases contraction in a canine post-infarction model. A: Individual, steady-state cell shortening (top) and Ca^2+^ transient (bottom) traces measured in post-MI canine cardiac myocytes with and without EMEPO incubation. B: Summary data (mean±s.e.m.) of the effect of EMEPO on shortening (left) and Ca^2+^ transient (right) amplitudes C: Summary data (mean±s.e.m.) of the effect of EMEPO on rate of relaxation (left) and [Ca^2+^]_i_ decline (right) measured as time to 50% relaxation (RT_50_). * P<0.05 vs. -EMEPO. n = 14–20 myocytes/2 hearts per group.

We extended our evaluation of EMEPO to a pathological model. We chose our well-characterized post-MI canine model [29]. This model was chosen since these hearts have redox-mediated changes in Ca^2+^ handling [30]. As expected, EMEPO increased contraction and relaxation rates in myocytes isolated from post-MI canine hearts with no effect observed in myocytes from control canine hearts (Figure 7). Similar to the NOS1^−/−^ myocyte data, post-MI myocytes incubated with EMEPO significantly exceeded contraction (both shortening and Ca^2+^ transient amplitudes) measured in control myocytes. Hence, our data suggest that EMEPO is effective to increase contraction by improving Ca^2+^ handling in diseased myocytes (i.e., post-MI). These data also suggest that EMEPO can be effective in larger mammalian species.

In summary, our results suggest that an imbalance of O_2_^.−^ and NO levels causes abnormal myocyte function. Concurrent restoration of O_2_^.−^ and NO levels will restore myocyte function via enhanced SR Ca^2+^ handling Thus, restoring both NO and O_2_^.−^ levels to reestablish the nitroso-redox equilibrium may prove useful in the treatment of various cardiomyopathies.

## Supporting Information

Text S1 (DOCX)
