# Supplementary material for: Diesterified Nitrone Rescues Nitroso-Redox Levels and Increases Myocyte Contraction Via Increased SR Ca2+ Handling
Source: PLoS One. 2012 Dec 27;7(12):e52005. doi: 10.1371/journal.pone.0052005 (PMC3531448; doi:10.1371/journal.pone.0052005)
Supplement: Text S1 — (DOCX) [file pone.0052005.s001.docx]

**Supplementary Text S1**

**Diesterified nitrone recovers nitroso-redox levels and increases myocyte contraction via increased SR Ca^2+^ handling**

Christopher J Traynham^1^, Steve R Roof^1^, Honglan Wang^1^, Robert A Prosak^2^, Lifei Tang^1^, Serge Viatchenko-Karpinski^1^, Hsiang-Ting Ho^2^, Ira O Racoma^2^, Dominic J Catalano^1^, Xin Huang^1^, Yongbin Han^2^, Shang-U Kim^2^, Sandor Gyorke^1^, George E Billman^1^, Frederick A Villamena^2^*, Mark T Ziolo^1^*.

* Authors contributed equally

Short Title: Traynham Restoring NO/O_2_^.-^ improves contraction

**Detailed Methods:**

**Synthesis of 5-(2-Ethoxy-2-oxoethyl)-5-(ethoxycarbonyl)-1-pyrroline *N*-oxide (EMEPO)**

The synthesis of EMEPO is shown in Scheme 1 according to the procedure previously described^1^. The synthesis of diethyl 2-nitrosuccinate (3) from 1 and 2 was carried out according to published procedures^2, 3^ and its spectral data was in agreement with the literature.^2, 3^ TEA (0.2 mL) was slowly added to a cooled (10 ^o^C) solution of 7 (1.8 g, 8.2 mmol/L) and acrolein (0.66 mL, 9.9 mmol/L) in CH_3_CN (5mL). The mixture was stirred for 2 hrs at room temperature and then concentrated under reduced pressure to give the crude product 4. The crude product 4 was dissolved in THF (90 mL) at -10 ^o^C bath and a solution of NH_4_Cl (2.8 g) in 20 mL water was added followed by Zn dust (2 g) which was added within 0.5 hr portion-wise at -10 ^o^C. The mixture was stirred for another 2 hrs at -10 ^o^C, then solid NaCl was added until saturated and extracted with Et_2_O. The collected organic phase was dried over anhydrous MgSO_4_ and filtered. The solvent was removed *in vacuo* and the residue was purified by flash column chromatography using CH_2_Cl_2_/MeOH (97:3 v/v) as eluent to give EMEPO as yellow oil (1.2 g, 60%). ^1^H NMR (400 MHz, CDCl_3_) δ 1.29 (overlap of two triplets, 6H), 2.71 (m, 1H), 2.76 (m, 3H), 3.05-3.33 (AB system, 2H), 4.13-4.27 (m, 4H), 6.97 (t, 1H). ^13^C NMR (100 MHz, CDCl_3_) δ 14.3, 14.5, 26.9, 30.2, 38.0, 61.3, 63.0, 79.9, 136.3, 169.1, 170.0. IR (neat, cm^-1^) *ν* 3440, 2983, 1733, 1586, 1207, 1183, 1076, 1027. GC-MS calcd for C_11_H_17_NO_5_ *m/z* 243.1, found 243.0. HRMS calcd for (M+Na) C_11_H_17_NO_5_Na *m/z* 266.1004, found 266.0995.

All the animal protocols and procedures were performed in accordance with National Institutes of Health guidelines and approved by the Institutional Laboratory Animal Care and Use Committee at The Ohio State University.

**Myocardial Infarction Procedure**

As previously described, heartworm free mongrel dogs were surgically administered a myocardial infarction ^4, 5^. Briefly, 24 hrs before surgery, a transdermal fentanyl patch that delivers 100 μg/hr (Duragesic, Jansen Pharmaceutica, Titusville, NJ, USA) was placed on the left side of the animal’s neck and secured with tape. On the day of surgery, the dogs received 15 mg (1 ml, i.m.) morphine sulfate (Elkins-Sinn, Cherry Hill, NJ, USA) and thiopental sodium (Baxter Healthcare, Glendale, CA, USA; 20 mg/kg, i.v.) to induce anesthesia. The dogs were intubated and a surgical plane of anesthesia was maintained by the inhalation of isoflurane (1–1.5%, Baxter Healthcare, Glendale, CA, USA). Using strict aseptic procedures, a left thoracotomy was made in the fourth intercostal space. The heart was exposed and supported by a pericardial cradle. The left circumflex coronary artery was dissected free of the surrounding tissue. A hydraulic vascular occluder (model OC3; In Vivo Metric, Healdsburg, CA) was then placed around this vessel. A two-stage occlusion of the left anterior descending artery was then performed approximately one-third the distance from its origin in order to produce an anterior wall myocardial infarction. This vessel was partially occluded for 20 min and then tied off.

**Mouse Cardiac Myocyte Isolation**

As previously described, myocytes were isolated from 4-5 month old NOS1 Knockout (NOS1^-/-^) and their corresponding wild-type (WT, C57BL/6J) (Jackson Laboratories, Bar Harbor, Maine) mice^6^. Mice were anesthetized with sodium pentobarbital (i.p. injection of 50mg/kg). Hearts were cannulated and perfused on a Langendorff apparatus at 37°C with Ca^2+^ free normal Tyrode solution (in mmol/L: 140 NaCl, 4 KCl, 1 MgCl_2_, 10 Glucose, 5 HEPES, pH 7.4) for four minutes. After which time, the flow was switched to tyrode solution containing Liberase Blendzyme IV (Roche Diagnostics) and 50 μmol/L CaCl_2_. Digestion with Blendzyme IV lasted three to five minutes. The heart was removed from the Langendorff, atria trimmed off, and the heart minced. Tissue was triturated, filtered and spun down to produce cell pellets. The pellets were washed with 50 μmol/L CaCl_2_ Tyrode and re-suspended in normal Tyrode solution supplemented with 2 X 10^-4^ mol/L CaCl_2_. Isolated myocytes were used experimentally within 4-6 hours of their isolation.

**Canine Cardiac Myocyte Isolation**

In brief, ten weeks following the surgery and MI, 2 MI dogs and 2 control dogs (weight: 21.2 ± 2.6 kg; age 2.0 ± 0.7 y) were anesthetized and the heart was excised. Septum chunks were harvested and placed in Cardioplegia solution (in mmol/L: 140 NaCl, 25 KCl, 1 MgCl_2_, 10 glucose, 5 HEPES, pH 7.4). Chunks were minced and digested with Collagenase Type II (307.5 units/mL, Worthington Biochemical Corporation, Lakewood, NJ) in a minimum essential medium eagle (MEM) enzyme solution that consisted of (in mmol/L): 2.4 NaHCO_3_, 3.4 MgCl_2_, 10 taurine, 0.009 BSA, 0.0008 insulin, 2 pyruvic acid, 0.005 trypsin, 0.05 CaCl_2_, aerated with 95 O_2_/5% CO_2_, pH 7.35. Myocytes were dissociated by trituration. Subsequently, the myocytes were filtered, centrifuged, and resuspended in tyrode storage solution containing 1 mmol/L Ca^2+^. Myocytes were used within 6 hrs of isolation.

**Measurement of O_2_^.-^ Levels**

Lucigenin-enhanced chemiluminescence was used to measure O_2_^.-^ levels in cardiac myocyte homogenates from WT and NOS1^-/-^ mice, as previously described^7^. Isolated myocytes were homogenized in Homogenization Buffer (in mmol/L): 1 Tris pH 7.5, 4 NaCl, 0.1 Sodium Pyrophosphate, 1 Sodium vanadate, 1 Benzamidine, 0.1 M NaF, 100% Tergitol NP-40). The homogenates were transferred into an opaque 96-well microplate containing 5 µmol/L lucigenin (bis-N-methylacridinium nitrate). Chemiluminescence was recorded by a Centro XS^3^ LB 960 luminometer (Berthold Technologies, Bad Wildbad, Germany) reporting relative light units (RLU) emitted over a 30-min period. O_2_^.-^ levels were reported as the chemiluminescence of lucigenin-containing buffer with tissue minus background. O_2_^.-^ production was expressed as RLU per µg/ml of protein.

**Measurement of NO levels**

Intracellular NO imaging was performed using an Olympus Fluoview 1000 laser scanning confocal microscope equipped with Olympus oil objective (60X, 1.4 NA). NO levels were measured in isolated cardiac myocytes from NOS1^‑/-^ and WT mice. Myocytes were incubated concurrently with DAF-FM diacetate (10 µmol/L, Molecular Probes, Eugene, OR) and EMEPO for 20 min and then 20 min was allowed for de-esterification. This is the only time myocytes were exposed to EMEPO. DAF-FM diacetate was excited at the 488nm line of an argon ion laser, and fluorescence was acquired at >510 nm in the X-Yscan mode of the confocal system at the rate of 5.2 s per scan. Maximum fluorescence, as a measure of myocyte dye loading, was obtained by superfusing the myocyte with 1 mmol/L Spermine NONOate (NO donor). The magnitude of fluorescent signals was quantified in terms of average pixel intensity. Data were expressed as the % of maximum DAF fluorescence (i.e. the ratio of basal DAF fluorescence divided by Spermine NONOate DAF fluorescence multiplied by 100).

**Simultaneous measurement of Ca^2+^ transients and myocyte shortening**

Simultaneous measurement of Ca ^2+^ transients and myocyte shortening was performed as previously described^6^. Myocytes were loaded at 22 °C with Fluo-4 AM for 30 min, washed out with either 2 X 10^-4^ mol/L CaCl_2_ normal Tyrode or 2 X 10^-4^ mol/L CaCl_2_ normal Tyrode supplemented with EMEPO (1 mmol/L), and then 30 min was allowed for intracellular de-esterification. This is the only time myocytes were exposed to EMEPO. The instrumentation used for cell fluorescence measurement was a Cairn Research Limited (Faversham, UK) epifluorescence system. [Ca^2+^]_i_ was measured by Fluo-4 epifluorescence with excitation at 480±20 nm and emission at 535±25 nm. The illumination field was restricted so the emission of a single cell could be collected. Data was expressed as ΔF/F_0_, where F is the fluorescence intensity and F_0_ is the intensity at rest. Mouse myocytes were field stimulated at 1 Hz via Grass S48 stimulator (West Warwick, RI, USA), while canine myocytes were field stimulated at 0.5 Hz. All experiments were performed at room temperature (22°C). Simultaneous measurement of shortening was performed using an edge detection system (Crescent Electronics, Sandy, UT). Data were expressed as % resting cell length (%RCL).

**Force-frequency experiment**

Force-frequency experiments were performed in isolated myocytes from NOS1^-/-^ and WT mice. Data were obtained in myocytes stimulated at 0.2, 0.5, and 1 Hz at room temperature.

**Measurement of SR Ca^2+^ leak/load relationship**

SR Ca^2+^ leak was measured as the tetracaine (RyR2 inhibitor)-induced shift in diastolic [Ca^2+^]_i_ ^8-10^. Cells were stimulated for 1 min in normal Tyrode solution. After reaching steady state, the solution was rapidly switched to 0 Na^+^, 0 Ca^2+^ Tyrode solution (Na^+^ was replaced by Li^+^) plus 1 mmol/L tetracaine (Sigma) for 30 s. Afterwards, the solution was rapidly switched to 0 Na^+^, 0 Ca^2+^ Tyrode solution for 20 s. Tetracaine, by blocking RyR2 and therefore SR Ca^2+^ leak, caused a decrease in diastolic [Ca^2+^]_i_. The shift in diastolic [Ca^2+^]_i­_ upon the removal of tetracaine was used as a measure of RyR2-dependent SR Ca^2+^ leak. Since SR Ca^2+^ leak is also dependent upon SR Ca^2+^ load, SR Ca^2+^ load of each cell was measured via rapid application of caffeine (10 mmol/L). Measurements were performed at room temperature.

**Western blot**

Western blots were performed on cardiac myocyte homogenates, as previously described ^6^. Total PLB and phoshporylated PLB were measured by using antibodies to PLB (Zymed, Invitrogen; Carlsbad, CA) and phophorylated PLB at serine16 (Badrilla, Leeds, UK).

**Solutions and Drugs**

1 mmol/L Ca^2+^ was used for mouse functional experiments and measurement of NO, while 2 mmol/L Ca^2+^ was used for canine functional experiments. DMPO (1 mmol/L, extracellular nitrone spin trap), EMEPO, ISO (1 µmol/L, non-specific β-AR receptor agonist), MENO (5 µmol/L, O_2_^.-^ scavenger), SNAP (1 µmol/L, NO donor) and Spermine NONOate were prepared fresh each experimental day.

**Statistics**

Results were expressed as mean ± s.e.m. Statistical significance (p<0.05) was determined by ANOVA (followed by Neuman-Keuls test) for multiple groups. Student t-tests were used for comparison between two groups.

**References:**

**1.** Han, Y, Liu, Y, Rockenbauer, A, Zweier, JL, Durand, G and Villamena, FA. Lipophilic beta-cyclodextrin cyclic-nitrone conjugate: synthesis and spin trapping studies. J Org Chem. 2009;74:5369-5380.

**2.** Diez-Barra, E, de la Hoz, A and Moreno, A. Alkylation of ethyl nitroacetate in the absence of solvent. Syn. Commun. 1994;24:1817-1821.

**3.** Schipchandler, MT. The utility of nitroacetic acid and its esters in organic synhtesis. Synthesis. 1979;9:666-686.

**4.** Belevych, AE, Terentyev, D, Viatchenko-Karpinski, S, Terentyeva, R, Sridhar, A, Nishijima, Y, Wilson, LD, Cardounel, AJ, Laurita, KR, Carnes, CA, Billman, GE and Gyorke, S. Redox modification of ryanodine receptors underlies calcium alternans in a canine model of sudden cardiac death. Cardiovasc Res. 2009;84:387-395.

**5.** Billman, GE. A comprehensive review and analysis of 25 years of data from an in vivo canine model of sudden cardiac death: implications for future anti-arrhythmic drug development. Pharmacol Ther. 2006;111:808-835.

**6.** Wang, H, Kohr, MJ, Traynham, CJ, Wheeler, DG, Janssen, PM and Ziolo, MT. Neuronal Nitric Oxide Synthase Signaling within Cardiac Myocytes Targets Phospholamban. Am J Physiol Cell Physiol. 2008;294:C166-C1575.

**7.** Khan, SA, Lee, K, Minhas, KM, Gonzalez, DR, Raju, SV, Tejani, AD, Li, D, Berkowitz, DE and Hare, JM. Neuronal nitric oxide synthase negatively regulates xanthine oxidoreductase inhibition of cardiac excitation-contraction coupling. Proc Natl Acad Sci U S A. 2004;101:15944-15948.

**8.** Knollmann, BC, Chopra, N, Hlaing, T, Akin, B, Yang, T, Ettensohn, K, Knollmann, BE, Horton, KD, Weissman, NJ, Holinstat, I, Zhang, W, Roden, DM, Jones, LR, Franzini-Armstrong, C and Pfeifer, K. Casq2 deletion causes sarcoplasmic reticulum volume increase, premature Ca2+ release, and catecholaminergic polymorphic ventricular tachycardia. J Clin Invest. 2006;116:2510-2520.

**9.** Picht, E, DeSantiago, J, Huke, S, Kaetzel, MA, Dedman, JR and Bers, DM. CaMKII inhibition targeted to the sarcoplasmic reticulum inhibits frequency-dependent acceleration of relaxation and Ca2+ current facilitation. J Mol Cell Cardiol. 2007;42:196-205.

**10.** Shannon, TR, Ginsburg, KS and Bers, DM. Quantitative assessment of the SR Ca2+ leak-load relationship. Circ Res. 2002;91:594-600.
